# Evaluation of an online modular eating disorders training (PreparED) to prepare healthcare trainees: a survey study

**DOI:** 10.1186/s12909-023-04866-1

**Published:** 2023-11-16

**Authors:** Deborah R. Glasofer, Diana C. Lemly, Caitlin Lloyd, Monica Jablonski, Lauren M. Schaefer, Stephen A. Wonderlich, Evelyn Attia

**Affiliations:** 1 New York State Psychiatric Institute, Columbia University Irving Medical Center, New York, USA; 2 Massachusetts General Hospital, Harvard University School of Medicine, Boston, USA; 3 Center for Biobehavioral Research, Sanford Research, Fargo, USA; 4 Department of Psychiatry and Behavioral Science, University of North Dakota School of Medicine and Health Sciences, Fargo, USA; 5 Weill Cornell Medical College, New York, USA

**Keywords:** Diagnosis, Education, Medical, Nursing, Nutrition, Technology, Training, Eating disorders

## Abstract

**Background:**

Eating disorders (EDs) are serious, complex disorders for which broad-based clinical training is lacking. This study aimed to evaluate the efficacy of a free, brief, web-based curriculum, PreparED, in increasing comfort and confidence with, and knowledge about EDs in healthcare trainees, and to obtain program feedback from key stakeholders (i.e., learners).

**Methods:**

This programmatic evaluation study was designed as a quantitative, repeated measures (i.e., pre- and post-test intervention) investigation. A convenience sample of two groups of healthcare trainees across geographically diverse training sites completed an anonymous survey pre- and post- engagement with PreparED. The survey included items to assess prior exposure to EDs, as well as program feasibility. The main educational outcomes included (1) Confidence and Comfort with EDs and (2) Knowledge of EDs. User experience variables of interest were likeability, usability, and engagement with the training modules. Mixed effects linear regression was used to assess the association between PreparED and educational outcome variables.

**Results:**

Participants (N = 67) included 41 nutrition graduate students and 26 nurse practitioner students recruited from Teacher’s College/Columbia University in New York, NY, USA, Columbia University School of Nursing in New York, NY, USA and North Dakota State University School of Nursing in Fargo, ND, USA. Confidence/Comfort scores and Knowledge scores significantly improved following engagement with PreparED (β = for effect of intervention = 1.23, p < 0.001, and 1.69, p < 0.001, respectively). Neither training group nor prior exposure to EDs moderated the effect on outcomes. All learners agreed the program was easy to follow; the overwhelming majority (89.4%) felt the length of the modules was “just right.” All participants perceived that PreparED had increased their knowledge of EDs, and the majority (94.0%) reported greater confidence in and comfort with caring for people with these disorders, including assessment of symptoms, awareness of associated medical complications, and likelihood of future screening.

**Conclusions:**

Findings suggest that brief, user-friendly, online courses can improve knowledge and attitudes about EDs, filling a critical gap in healthcare training.

**Supplementary Information:**

The online version contains supplementary material available at 10.1186/s12909-023-04866-1.

## Background

As a group, eating disorders (EDs) are defined by the presence of a significant disturbance in eating behavior associated with a clear impact on physical, psychological, or social functioning [1]. EDs affect up to 4% of the population, have serious implications on morbidity and mortality [2, 3] and are associated with a remarkably high global burden of disease across low-, middle-, and high-income countries [4]. Several features of EDs, such as the shame and secrecy that accompanies behaviors of illness [5] and the medical complications that occur secondarily [6, 7], influence the likely access points for individuals to be screened and treated [8–11]. Early identification and intervention of EDs are associated with improved outcomes [12, 13]; however, affected individuals may be more likely to present with complaints or concerns to a primary care practitioner, medical specialist, or dietician than a mental health provider more familiar with these disorders.

Historically, opportunities for ED training and ongoing education have been limited globally and consequently, many healthcare professionals find themselves wanting for more confidence in, comfort with, and knowledge about these disorders. For example, in a national UK medical school survey, less than two hours was spent on teaching about EDs on average and 20% of medical schools reported that they did not cover the topic at all [14]. A US survey of internal medicine, pediatrics, family medicine, and psychiatry (including child and adolescent psychiatry) found that 80% of the residency programs did not offer ED rotations, 14.5% provided no formal ED didactics, and those programs that did offer didactics did so minimally [15]. While surveys of a similar scope are lacking in the fields in nursing and nutrition/dietetics, existing reports indicate that students and professionals in these fields do not feel as confident, comfortable, or knowledgeable as they would like to be in EDs based on their training [16–18]. An Australian study of nutrition students and recent graduates found that with regard to ED diagnosis, the majority of respondents were not confident in their ability (77–84%, depending on specific diagnosis) and over 95% of participants felt they required further training [16]. Similar themes (i.e., difficulty with accurate diagnosis, perceived need for continuing education in EDs) are echoed in the literature on nurses [17, 19].

Across disciplines, limited coverage of ED education during training can have downstream effects on provider comfort and self-confidence to assess and appropriately refer patients with EDs, attitudes towards patients, and patient safety [18–21]. Moreover, as rates of patients seeking services for EDs increased globally during the COVID-19 pandemic [22–26], the need to fill this education gap is critical. Making ED training accessible to those who will be on the proverbial ‘frontlines’ of detection may improve identification of cases, diagnosis, monitoring, and referrals.

Research to date has shown that a little knowledge can go a long way to bolster providers’ comfort with and attitudes towards EDs, thereby enhancing patient care [20, 27]. The relative lack of ED education in healthcare training, well documented across disciplines [14–18], may in part be attributable to the paucity of ED experts who can provide supervision to trainees at varied geographic locations (e.g., rural communities) and across training disciplines [15]. Advances in technology offer a way to overcome these barriers. Even before the COVID-19 pandemic made remote learning a necessity, e-delivery of educational content was gaining popularity and several innovative efforts leveraged technology to improve access to ED education. For example, a one-hour asynchronous video training has been shown to improve screening and referral in pediatric primary care providers [27], and a comprehensive online ED education program (5 modules offering 15–20 hours of content) has provided a way to reach a wide range of practitioners, including those working in rural areas [28].

In this study, we aimed to evaluate the efficacy of a freely available web-based curriculum, PreparED, in improving confidence and comfort with, and knowledge about EDs in healthcare trainees. We hypothesized that this brief, modular training would improve outcomes of interest in two distinct trainee groups – nurse practitioner students and graduate students in nutrition. We also sought out user feedback on the program to explore likability, usability, and engagement by evaluating learners’ impressions of the curriculum’s length, coherence, and self-perceived impact on confidence, comfort, and knowledge.

## Methods

This programmatic evaluation research was designed as a single group, quantitative, repeated measures (i.e., pre- and post-test intervention) study.

### Recruitment

A convenience sample of students in two distinct healthcare disciplines (i.e., master’s level nutrition graduate students, nurse practitioner students) was recruited between April 2021 and May 2022. Nutrition graduate students were recruited from Teacher’s College/Columbia University in New York NY, USA. Nurse practitioner students were enrolled at either Columbia University School of Nursing in New York NY, USA or North Dakota State University School of Nursing in Fargo ND, USA. Prior to any coursework on eating disorders, 105 students received a letter (Appendix A) inviting them to participate in an anonymous survey to evaluate PreparED, an online ED educational tool, https://prepared.nyspi.org/ [29]. Participation was voluntary and no identifying information was collected. All participants were informed that their responses would be used for research purposes. Because the study assessment collected no personal identification data, this study was reviewed by the New York State Psychiatric Institute (NYSPI) Institutional Review Board, considered non human subjects research (i.e., program evaluation) and therefore exempted from needing informed consent procedures (on April 2, 2021).

### Intervention design and development: PreparED

PreparED is an online educational tool with content aimed at the graduate-level learner and non-ED specialist health care providers. Content was developed to be appropriate for those with no or limited prior background or exposure to EDs. The multi-disciplinary team of creators consisted of (1) a senior faculty psychiatrist with over 3 decades’ experience in ED education, research and clinical care [EA], (2) a mid-career faculty clinical psychologist with 15 years’ experience in ED education, research, and clinical care [DRG], (3) a junior social worker with < 5 years’ experience in ED clinical care [NP, see Acknowledgments], and (4) a second year medical student with no ED exposure prior to his rotation with our program [SB, see Acknowledgments]. The design and development process aligned with the ADDIE (Analysis, Design, Development, Implementation, and Evaluation) model [30, 31] used to build effective eLearning courses.

In the Analysis phase, PreparED creators came together to share perceptions of the problem (i.e., the gap in training) and identify the content topics that might meet the basic needs of the broadest audience, which was clarified early on to be healthcare students and non-ED specialist providers.

**Fig. 1:**
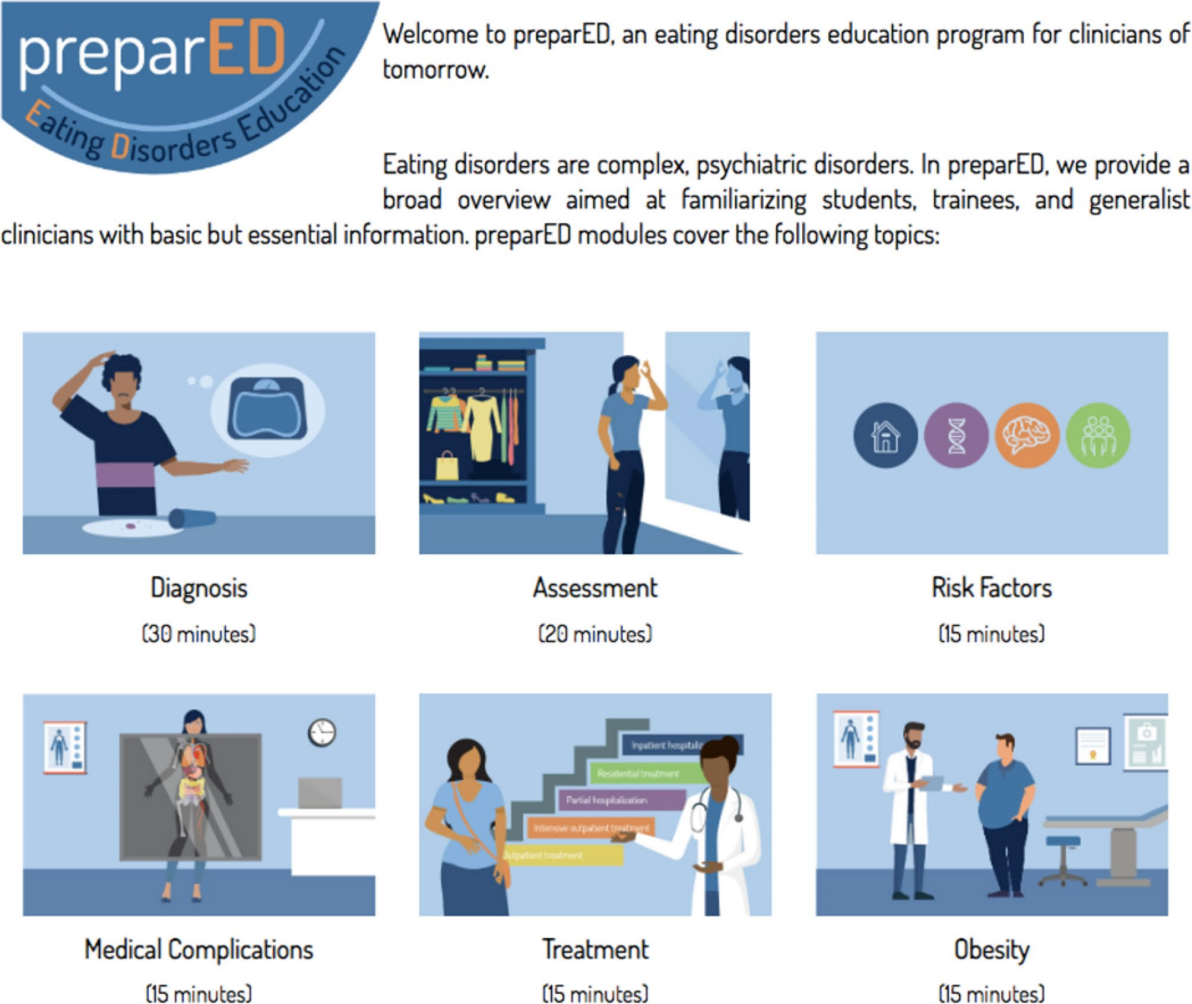
PreparED program modules. Freely available at https://prepared.nyspi.org

In the Design phase, eLearning consultants helped determine how best to package a curriculum so that it would be engaging and informative while placing minimal time burden on the learner. The program was intentionally designed to be freely available on an openly accessible website and to minimize user burden, use of the program does not require registration within a learning management system. A modular design was agreed upon. Ultimately, PreparED included six independent modules so that users can select the content that might best serve their educational needs (Fig. 1). Modules cover (1) diagnosis, (2) assessment, (3) medical complications, (4) treatment, (5) risk factors, and (6) obesity and EDs. Modules are brief (15–30 min each) with a total duration (i.e., “sit time”) of less than two hours. Modules were designed to be engaging, including animation and/or case material where relevant, and interactive, with quizzes (i.e., learning checks) embedded throughout. Each module provides access to a set of downloadable learning tools (e.g., medical assessment checklist, diagnostic checklist, first line treatment for EDs summary), as well as an educator’s guide with common questions and answers, and additional quiz questions to assess learning.

In the Development phase, course content was created drawing upon, adapting, and updating pre-existing material from co-creators’ [EA, DRG] experiences in education, including prior lectures given to healthcare students, conference presentations, and case consultations, as well as teaching materials provided by other ED faculty members who have been involved in education of medical students, nurses, residents, psychologists, social workers, and nutrition students over the last forty-plus years. Iterative feedback on design, scope of content, quizzes, and learning tools was provided by the healthcare trainee (medical student) program co-creator [SB, see Acknowledgments], as well as a range of other healthcare trainees and educators.

The Implementation phase started with a review of the educational program and initial, informal piloting with the newest members of an ED research team in 2020 and 2021. This included bachelors-level research assistants, medical student volunteers, and psychology externs, as they participated in other ED educational programming provided to onboard new staff. The current study is the next part of the Implementation phase and the beginning of the Evaluation phase.

### Measures: pre- and post- surveys

The PreparED Surveys (Appendix B) were developed by a multi-disciplinary team of ED experts (clinical psychologist, psychiatrist, and primary care physician) in line with Kirkpatrick’s four-level program evaluation model, covering the first two identified rungs of the theoretical hierarchy: (1) user feedback and (2) indicators of learning attributable to the program (in this case, changes in confidence, comfort and knowledge) [32, 33]. The learning outcomes selected were consistent with Miller’s taxonomy for assessing clinical competence including knowledge acquisition and integration of intellectual knowledge and professional attitudes to perform optimally as a health professional [34].

**Fig. 2:**
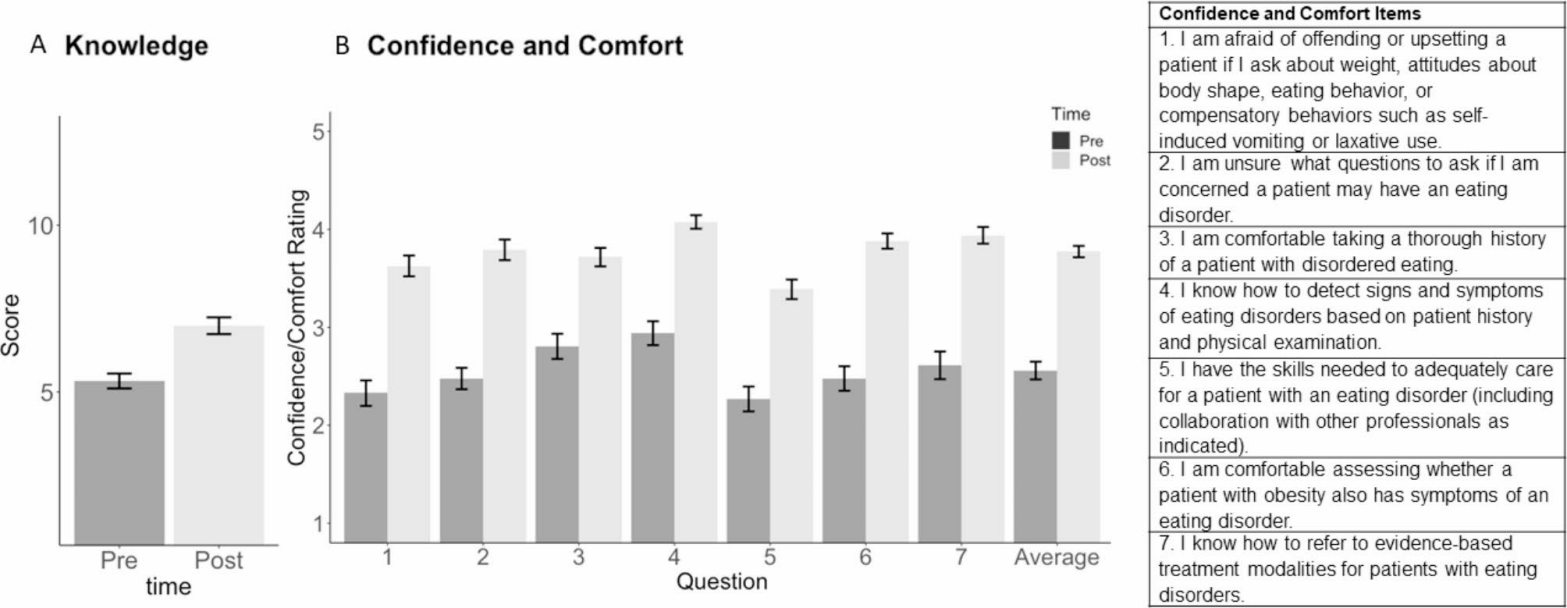
Effect of PreparED program on knowledge, confidence and comfort

The Pre-Survey included 3 items on prior Educational Experience with EDs including types of exposure to EDs in training (e.g., video, lecture, reading, clinical teaching), curriculum hours spent on ED education (i.e., None, < 1 h, 1–2 h, > 2 h), and clinical experience (i.e., care of a patient with an ED) if applicable. Confidence and Comfort statements related to EDs (7 items; Fig. 2 – items 2, 4, 5, and 7 assessing Confidence and items 1, 3, and 6 assessing Comfort) measured participants’ self-perceived ease in screening for EDs (e.g., “I am unsure what questions to ask if I am concerned a patient may have an eating disorder.”, “I am comfortable assessing whether a patient with obesity also has symptoms of an eating disorder.”) on a 5-point scale from *strongly disagree* to *strongly agree*. Two items with negative phrasing (e.g., “I am unsure…,” “I am afraid…”) were reversed scored. Therefore, higher scores indicate more Confidence and Comfort. The Pre-Survey also included 11 multiple choice Knowledge items on diagnosis (2 items), assessment (1 item), treatment (3 items), medical complications (2 items), obesity and EDs (2 items), and risk factors (1 item). Knowledge items were repeated in the Post-Survey, along with 7 additional items to provide feedback on curriculum length (“The time spent to complete PreparED was (a) too short, (b) just right, (c) too long.”), and using a 5-point scale from *strongly agree* to *strongly disagree*, on PreparED’s usability (e.g., “The curriculum was easy to follow.”), and self-perceived impact on confidence and comfort (e.g., “After completing the curriculum, I will be more likely to screen for eating disorders.”), as well as knowledge (e.g., “Completing this curriculum increased my knowledge of eating disorders.”).

### Analysis

All analyses were completed in Rstudio. Average agreement with the Confidence and Comfort scale was calculated: the negative items were reverse scored (i.e., a score of 5 was recoded as 1, etc.) and the mean rating across the 7 items was determined for each participant. To assess whether Confidence and Comfort scores improved following PreparED, a linear mixed effects regression was completed. The model specified fixed effects of time (i.e., pre- versus post-survey completion) and group (i.e., nutrition graduate student or nurse practitioner student). The model included an indicator variable that indexed prior exposure to EDs in a clinical or academic setting, and age, as fixed effect covariates. The analysis was repeated including a group x time interaction term to test whether there was a difference in Confidence and Comfort score improvement following PreparED between student groups. The main analysis was also repeated including an exposure x time interaction, to test whether prior exposure affected the change in Confidence and Comfort following PreparED. All statistical models included a random intercept for each participant.

To examine the efficacy of PreparED on Knowledge, a linear mixed effects regression was completed, specifying fixed effects of time and group and, again, including age and prior exposure to EDs as covariates. The analysis was repeated including a group x time interaction term to test whether there was a difference in learning outcomes between groups, and (separately) an exposure x time interaction, to test whether prior exposure affected the change in knowledge score following PreparED. Similar to the analyses of Confidence and Comfort scores, all statistical models involving Knowledge scores specified a participant-level random intercept. All mixed effects linear regression models met assumptions of linearity, homogeneity of variance, and normality of residuals. To determine the size of the effect of time, Cohen’s d was calculated.

## Results

### Participants

Participants (N = 67) included 41 nutrition graduate students (pursuing a Master’s degree) and 26 nurse practitioner students. This represented an overall response rate of 63.8%. Most of the sample (70.1%) was age 18–29, 26.9% of participants were age 30–39, and 3% of participants were over age 40. Because two individuals were missing information about prior ED training, the main analyses included 65 participants.

### ED exposure in training

Among nutrition graduate students, 28.9% reported no exposure in their curriculum to EDs. Two-thirds (66.6%) of those who had some form of ED curriculum (e.g., video, lecture, reading, clinical teaching) reported less than two hours spent on the topic.

In the nurse practitioner student group, the minority (11.5%) reported no exposure in their curriculum to EDs. For those who had some form of ED curriculum (e.g., video, lecture, reading, clinical teaching), 70.8% had spent less than two hours on the topic. 19% of the nurse practitioner trainees reported no exposure to information about EDs during their clinical rotations thus far, and 42.3% of this group had not participated in the care of an ED patient.

### PreparED’s impact on confidence and comfort

Outcomes of the primary linear mixed effects regression model (including no interaction terms) indicated that average Confidence and Comfort scores improved from pre-to-post survey. The mean Confidence and Comfort score was 2.56 (SD = 0.74) prior to PreparED and 3.77 (SD = 0.48) following the training (Fig. 2): β for effect of time = 1.23, 95% CI: [1.05, 1.41], p < 0.001.). This constituted a large effect of time: the effect size, as indexed Cohen’s d, was d = 1.34. Prior exposure was associated with improved Confidence and Comfort at both time points (β for prior exposure = 0.34, 95% CI: [0.07, 0.61], p = 0.014), though there was no effect of group on average ratings (β for group [graduate students as reference] = -0.05, 95% CI: [-0.34, 0.24], p = 0.726) Neither group nor exposure moderated the effect of PreparED training on score: β for group x time interaction = 0.03, 95% CI:[-0.34, 0.41], p = 0.864; β for exposure x time interaction = -0.30, 95% CI: [-0.69,0.09], p = 0.135).

### PreparED’s impact on knowledge

There was a significant effect of time on Knowledge, with scores increasing after engagement with PreparED (Fig. 2; Time 1 mean score = 5.33 (SD = 1.83), Time 2 mean score = 6.99 (SD = 2.06), β for effect of time = 1.69, 95% CI: [1.27–2.12], p < 0.001). Again, the effect of time was large: Cohen’s d = 1.02. Prior ED curriculum exposure was not associated with score at pre- and post- curriculum assessments (β = -0.05, CI: -1.00, 0.91, p = 0.923), and neither was group (β for group [graduate students as reference] = -0.97, 95% CI: [-1.99, 0.05], p = 0.062). Nurse practitioner students and nutrition graduate students did not differ in their score improvements pre- and post-curriculum (β for group x time interaction = -0.06, 95% CI: [-0.94, 0.81], p = 0.885). Prior exposure also did not affect the change in knowledge following PreparED: β for exposure x time interaction = -0.23, 95% CI: [-1.15, 0.70], p = 0.627.

Panel A shows the change in Knowledge following PreparED. Mean scores on the assessment were calculated and compared for pre- and post- engagement with the online training. Panel B shows the change in Confidence and Comfort following PreparED training. Items 1 and 2 were reversed scored so that higher values on the rating scale indicate greater comfort/confidence. Mean scores for each item, and for the participant average across all items, were calculated and compared at pre- and post- training.

### Feedback on intervention

Participants unanimously (100%) reported that PreparED increased their knowledge of EDs. Nearly all survey respondents perceived that the educational program increased their comfort with and confidence in caring for people with EDs (94.0% agreement), asking questions about symptoms (97.0% agreement), and recognizing associated medical complications (92.3% agreement). After engaging with PreparED, 88.1% of participants believed they were more likely to screen for EDs. All users (100%) agreed that the program was easy to follow, and the overwhelming majority (89.4%) felt the length of the modules was “just right” (rather than “too short” or “too long”).

## Discussion

Findings from this program evaluation survey suggest that brief, user-friendly, online courses can improve knowledge about and confidence and comfort with EDs. In this study, neither healthcare discipline nor prior exposure to EDs impacted the helpfulness of PreparED in improving confidence, comfort, and knowledge; all learners benefited from the program on all outcomes of interest.

Study participants provided unequivocally positive feedback on the curriculum and its design. The post-survey included items geared towards co-design, in which stakeholders – in this case, learners – are asked to provide feedback on a product’s design. Co-design methods are associated with improving user satisfaction or engagement [35], and may be especially important when combining the use of technology (i.e., e-learning) with clinical education [36]. Quantitative (e.g., feedback scales) and qualitative (e.g., focus groups) methods have been used to ensure that curriculums and e-learning resources are student-centered and aligned to the learners’ expectations and needs in medical and nursing education [37, 38]. Because stakeholder analysis during the brainstorming and editing stage is a co-design method suggested to be critical in creating eLearning products [39], a medical student was invited to help develop, design, and refine PreparED. Given the substantial curricular demands for healthcare trainees, which make it difficult to add material, feedback on the program’s duration was essential. Here, nearly 90% of study participants rated the sit time of the modules as “just right,” suggesting that other trainee groups may find the program’s use feasible and its demands reasonable. Findings like these, alongside advocacy by ED experts, are necessary to effect lasting and systemic educational change. For this kind of change to occur, future research might also include educators across healthcare disciplines as stakeholders.

Efforts to promote the importance of ED education ought to provide information about the high levels of associated morbidity and mortality [2, 3], the likelihood that patients with these disorders present to emergency departments [6, 8] and a range of general health settings [9, 10, 40], and that better outcome is associated with early identification and treatment [12, 13, 41]. Supplying data on the substantial increases in ED cases presenting to hospitals and other settings during the COVID-19 pandemic [25, 42, 43] may underscore the need for current health care providers (in addition to trainees) to improve competency in ED evaluation.

The post-survey also assessed changes in self-efficacy (i.e., belief in one’s abilities and capacities). Self-efficacy beliefs enable a sense of agency and can play a unique role in motivating behavior, including the actions of healthcare providers. For example, following a training on suicide risk management, increased self-efficacy was linked to changes in clinicians’ attitudes about suicidality *and* suicide prevention practices [44]. In this study, over 90% of our sample perceived that PreparED helped them develop more confidence in diagnosis and assessment, and increased their recognition of medical complications associated with EDs. This aligned with positive, significant changes in pre-post confidence and comfort ratings on all items, as well as improvements in knowledge scores. Taken together, our results suggest that a brief educational tool can improve both likelihood and ability to screen for these conditions.

Attitude towards ED patients is another key contributor in screening, diagnosis, referral, and ongoing care. To date, the literature suggests that many providers view patients with EDs in a negative manner and as somewhat responsible for their condition [19, 21]. Preconceived beliefs about patients with EDs in concert with key features of the disorders themselves, such as secrecy and denial of the seriousness of certain symptoms, may result in the provider experience of frustration, anger, or helplessness [10, 17]. In this study, greater prior exposure to EDs in training was associated with more comfort with this patient population, though the educational intervention was beneficial for all learners. Prior research has suggested that improvement in ED knowledge and training may impact attitudes and towards caring for this patient population and related behaviors [19]. It is also possible that curriculum product design, in addition to content, has the potential to dispel pervasive stereotypes or implicit biases about EDs which can contribute to delays in diagnosis [13, 45, 46], referral [47], and disparities in access to care [48]. The design aesthetic selected for PreparED was done so with intention, including images of people with a range of skin tones and body sizes, and using case material with examples of EDs in boys and men. Future study is required to determine the effect of ED education on attitudes and biases towards the patient population, to ensure that educational efforts are maximizing their impact, and to test for changes in case identification.

ED education can be delivered in several ways. Material can be covered in traditional didactics, through readings, videos, and podcasts, via clinical exposure (i.e., planned or elective rotations), or relying upon expert case consultation or ongoing supervision. A benefit to traditional didactics and readings is that content can be covered in a manner that may be ideally suited to ways in which some learners will be subsequently quizzed on the material (e.g., exams for board certification or professional licensure). Films and podcasts from the perspective of those with lived experience of EDs bring nuances of these disorders – and the wide range of people who experience them – to life, though it may be hard to generalize from one story to the next. Clinical exposure and teaching associated with it (whether rotations, consultations, or ongoing supervision) afford learners the opportunity to combine learning essential skills in assessment and diagnosis with appreciating a patient’s personal narrative *and* the chance to participate in clinical decision making (e.g., medical evaluation, referral, or ongoing care). Moreover, it is clear from the literature that a little bit of clinical exposure can go a long way in improving provider confidence, comfort, and knowledge [20]. However, the pool of ED experts to provide training or programs with ready access to the patient population is a limited resource and highly mismatched to the current need. Literature documenting the gap in training consistently recommends the leveraging of technology to address the problem via one-time webinars, online courses, or asynchronous learning tools [15]. This recommendation is echoed in survey studies of healthcare students and professionals, with respondents reporting a preference for online ED training [16, 18]. In addition to offering more equitable access to ED training, PreparED was designed to deliver essential information (as provided in an in-person didactic), assess learning with quiz questions akin to what a student might see on an exam, and offer case examples bring the nuances of these disorders and the fundamentals of clinical decision-making to life. This study of a freely available online resource adds to the growing literature [27, 28] suggesting that innovative ED education products may be effective.

Strengths of this study include use of traditional and co-design methodology, inclusion of multiple dimensions by which to evaluate a novel educational tool, and a geographically diverse sample. A main limitation is that data were collected only from trainees who chose to complete graduate level coursework in EDs and were willing to participate, and as such, their perspectives may not be fully representative of their trainee group. Selection bias may have contributed to a moderate pre-PreparED mean knowledge score and may explain why, in our sample, a minority of students reported no prior exposure to EDs in their training curriculum. Inclusion of only two student groups (i.e., clinical nutrition graduate students and nurse practitioner students) is another limitation. Notably, efforts by the authors to include medical students and primary care medical trainees were unsuccessful because programs were reluctant to add to curricular demands. This study is also limited by its lack of a no-intervention control group. Though a common limitation in experimental medical education research [49], it is important to acknowledge that definitive conclusions about the utility of a curriculum like PreparED might be best captured through inclusion of a control comparison group. Finally, this study is limited by a lack of identifying information from participants as this might have helped identify if subgroups benefit differentially from course offerings.

## Conclusion

In this study, all learners improved confidence and comfort with, and knowledge about EDs regardless of healthcare discipline or prior educational background. Moreover, results indicated that participants found the online program coherent and user-friendly and liked that the curriculum was brief. Identifying acceptable, feasible, accessible educational programs to help health professional students and non-specialized providers learn about EDs may help close the learning gap for frontline and future frontline providers who are often asked to evaluate individuals with these complex disorders. Given the increased numbers of cases presenting worldwide, the need is urgent. Fortunately, technology offers a pathway to increase accessibility of experts and by extension, availability of ED education. As we develop new educational tools, we should engage key stakeholders in their development and evaluation and rigorously study their influence on knowledge and attitudes to maximize their impact.

### Electronic supplementary material

Below is the link to the electronic supplementary material.

Supplementary Material 1

Supplementary Material 2

## Data Availability

The data supporting the results reported in this article are maintained by the Eating Disorders Research Unit of the New York State Psychiatric Institute. Please contact the corresponding author with data requests.

## List of abbreviations

**ED**: Eating disorders
**NYSPI**: New York State Psychiatric Institute
