# Supplementary material for: Evaluation of an online modular eating disorders training (PreparED) to prepare healthcare trainees: a survey study
Source: BMC Med Educ. 2023 Nov 16;23:868. doi: 10.1186/s12909-023-04866-1 (PMC10652638; doi:10.1186/s12909-023-04866-1)
Supplement: Supplementary file 1 — Supplementary Material 1 [file 12909_2023_4866_MOESM1_ESM.docx]

**APPENDIX B.**

**PreparED Evaluation Survey**

**Prior Exposure/Educational Background for EDs – Pre-Survey only**

1. In your **graduate student curriculum**, did you have any of the following exposure to information about eating disorders?
   1. Watched a video or film (Y/N)
   2. Attended a lecture (Y/N)
   3. Standardized patient experience (Y/N)
   4. Assigned reading (Y/N)
   5. Clinical experience caring for a patient with a diagnosed eating disorder (Y/N)
   6. Explicit clinical teaching from faculty about a patient with an eating disorder (Y/N)
   7. None (Y/N)
   8. Other (Y/N)
2. In total, how much time **during your graduate student curriculum** did you spend learning about eating disorders?
   1. None
   2. < 1 hour
   3. 1-2 hours
   4. > 2 hours

3. As part of your graduate student curriculum, do you complete clinical rotations?

1. Yes
2. No

IF YES to clinical rotations question: During **clinical rotations** *(so far)*, did you have any of the following exposure to information about eating disorders?

1. Watched a video or film (Y/N)
2. Attended a lecture (Y/N)
3. Standardized patient experience (Y/N)
4. Assigned reading (Y/N)
5. Independent reading (Y/N)
6. Clinical experience caring for a patient with a diagnosed eating disorder (Y/N)
7. Explicit clinical teaching from faculty about a patient with an eating disorder (Y/N)
8. None (Y/N)
9. Other (Y/N)

IF YES to clinical rotations question: In total (so far), how much time **during clinical rotations** have you spent learning about eating disorders?

- 1. None
  2. <1 hour
  3. 1-2 hours
  4. > 2 hours

**Confidence and Comfort Items – Pre- and Post-Survey**

**To what degree do you agree with the following statements?**

*( 1= strongly disagree 2= disagree 3=neither agree nor disagree 4= agree 5=strongly agree, 6= not applicable)*

1. I am afraid of offending or upsetting a patient if I ask about weight, attitudes about body shape, eating behavior, or compensatory behaviors such as self-induced vomiting of laxative use.
2. I am unsure what questions to ask if I am concerned a patient may have an eating disorder.
3. I am comfortable taking a thorough history of a patient with disordered eating.
4. I know how to detect signs and symptoms of eating disorders based on patient history and physical examination.
5. I have the skills needed to adequately care for a patient with an eating disorder (including collaboration with other professionals as indicated).
6. I am comfortable assessing whether a patient with obesity also has symptoms of an eating disorder.
7. I know how to refer to evidence-based treatment modalities for patients with eating disorders.

**Knowledge Items – Pre- and Post-Survey (correct answers in bold)**

*Please answer the following questions to the best of your ability.*

1. Which of the following psychiatric disorders is associated with the highest mortality rate? [DIAGNOSIS]

A. Bulimia Nervosa

B. Schizophrenia

**C. Anorexia Nervosa**

D. Bipolar Disorder

1. Asking about compensatory behaviors aimed at preventing weight gain may do all of the following except to: [ASSESSMENT]

**A. Encourage the patient to start using these behaviors**

B. Inform diagnosis of an eating disorder

C. Provide an opportunity to educate the patient on the risks of compensatory behaviors

D. Guide further medical assessment

1. Which is the most common eating disorder? [DIAGNOSIS]

A. Bulimia Nervosa

B. **Binge-Eating Disorder**

C. Anorexia Nervosa

D. Avoidant Restrictive Food Intake Disorder

4. Antidepressants have demonstrated efficacy for individuals with which of the following eating disorders? (select all that apply) [TREATMENT]

A. Anorexia Nervosa

**B. Bulimia Nervosa**

**C. Binge-eating Disorder**

D. Avoidant Restrictive Food Intake Disorder

5. Individuals with anorexia nervosa may develop all of the following physiological complications (select all that apply): [MEDICAL COMPLICATIONS]

A. High white blood cell count (leukocytosis)

**B. Increased liver function tests**

**C. High cholesterol**

**D. Decreased brain mass**

6. Which of the following is **not** a medical complication associated with bulimia nervosa? [MEDICAL COMPLICATIONS]

A. Dental erosion

B. Electrolyte disturbances

**C. QTc Prolongation**

D. Esophageal tears

7. The psychotherapies with a strong evidence base for the treatment of certain eating disorders are (select all that apply): [TREATMENT]

A. Interpersonal therapy (IPT) (for Anorexia Nervosa)

**B. Enhanced Cognitive Behavioral Therapy (CBT-E) (for Binge-Eating Disorder)**

**C. Family Based Therapy (FBT) (for Anorexia Nervosa)**

D. Dialectical Behavioral Therapy (DBT) (for Bulimia Nervosa)

8. The only **FDA-approved** medications for the treatment of certain eating disorders are (select all that apply): [TREATMENT]

A. Olanzapine (for Anorexia Nervosa)

**B. Lisdexamfetamine (for Binge-eating Disorder)**

**C. Fluoxetine (for Bulimia Nervosa)**

D. Sertraline (for Avoidant Restrictive Food Intake Disorder)

9. An individual with obesity may have the following eating disorder diagnoses (select all that apply): [OBESITY]

**A. Atypical Anorexia Nervosa**

**B. Binge-Eating Disorder**

**C. Bulimia Nervosa**

**D. Night Eating Syndrome**

1. Select the true statement(s) below: [OBESITY]
   - 1. Obesity is a psychiatric disorder.
     2. Individuals who have obesity always meet criteria for Binge-Eating Disorder.
     3. **Obesity may be related to the treatment of other psychiatric disorders.**
     4. **Medical complications of atypical anorexia can, in some cases, be as severe as from classic anorexia nervosa.**

**User Feedback – Post-Survey only**

**How much do you agree with the following statements?**

*(1= strongly disagree 2= disagree 3=neither agree nor disagree 4= agree 5=strongly agree, 6 = Not applicable,)*

1. Completing the PreparED curriculum increased my knowledge of eating disorders.
2. Completing the PreparED curriculum increased my confidence care for patients with eating disorders.
3. The PreparED curriculum was easy to follow.
4. After completing the PreparED curriculum, I will be more likely to screen for eating disorders.
5. After completing the PreparED curriculum, I will be more comfortable asking detailed questions about disordered eating behaviors and cognitions.
6. After completing the PreparED curriculum, I am more confident that I will recognize medical complications of eating disorders.
7. The time spent to complete the PreparED curriculum was:
8. Too short
9. Just right
10. Too long
