# Supplementary material for: Evaluation of an online modular eating disorders training (PreparED) to prepare healthcare trainees: a survey study
Source: BMC Med Educ. 2023 Nov 16;23:868. doi: 10.1186/s12909-023-04866-1 (PMC10652638; doi:10.1186/s12909-023-04866-1)
Supplement: Supplementary file 2 — Supplementary Material 2 [file 12909_2023_4866_MOESM2_ESM.docx]

**APPENDIX A.**

**PreparED Sample Recruitment Letter**

**Impact of PreparED—An Online Educational Program About Eating Disorders**

Thank you for your interest in helping us evaluate PreparED, an online educational program about eating disorders, developed by the team at the Columbia Center for Eating Disorders and NY State Psychiatric Institute. Below are some instructions that you will need when you are completing the online surveys.

The link below contains **BOTH** the Pre and Post Curriculum Surveys…

…Eating disorders education has historically been limited in curricula for healthcare trainees yet these disorders, affecting up to 4% of the population, have serious implications on morbidity and mortality, constituting a significant public health burden. Aiming to fill this gap in training, we developed PreparED, a series of online modules covering topics related to eating disorders including: diagnosis, assessment, risk factors, medical complications, treatment, and the relationship to obesity.

To evaluate the program, we are seeking feedback from medical students, nursing students, medical residents, and other graduate-level learners such as nutrition students. **The Pre and Post Surveys will each take approximately 10 minutes.** You will be asked about your training background, previous educational and clinical experience with and exposure to eating disorders, and impressions of PreparED’s content and style.
